# Getting the word out: Methods of learning about research and motivations for participation in a study focusing on a reproductive-aged Latina/x population

**DOI:** 10.1017/cts.2022.10

**Published:** 2022-01-31

**Authors:** Nicole M. Sekula, Torie C. Plowden, Anne Waldo, Richard Bryce, Maricela Castillo-Mackenzie, Sonia Acosta, Felix Valbuena, Mercedes Carnethon, Erica E. Marsh

**Affiliations:** 1 Division of Reproductive Endocrinology and Infertility, Department of Obstetrics and Gynecology, University of Michigan Medical School, Ann Arbor, MI, USA; 2 Department of Gynecologic Surgery and Obstetrics, Womack Army Medical Center, Ft Bragg, NC, USA; 3 Community Health and Social Services (CHASS) Center, Detroit, MI, USA; 4 Department of Family Medicine, University of Michigan Medical School, Ann Arbor, MI, USA; 5 Centro Multicultural LA Familia (CMLF), Pontiac, MI, USA; 6 Department of Preventative Medicine, Northwestern University Feinberg School of Medicine, Chicago, IL, USA

**Keywords:** Community engagement, Latinx, diverse study populations, reproductive health, study recruitment and retention

## Abstract

**Background:**

Although one of the fastest-growing populations in the USA, Latinx individuals remain underrepresented in research. In this study, we aimed to identify how Latina/Latinx participants of the Environment, Leiomyomas, Latinas, and Adiposity Study (ELLAS) learned about the research study and what motivated them to participate.

**Materials and Methods:**

Using a standardized survey tool, bilingual staff interviewed participants and asked them, 1) how they heard about ELLAS and 2) to identify and rank their top three reasons for participating in ELLAS.

**Results:**

“Word of mouth” through a friend or relative was the most common method of learning about ELLAS (49.0%), followed by a “community outreach event” (29.3%). The three most common reasons for participating in ELLAS were “to learn more about women’s health” (83.3%), “to receive a free health assessment” (79.4%), and “to contribute to scientific knowledge” (59.5%). Correlation between demographic and socioeconomic characteristics and participant responses indicated that there are different reasons for participation based on these factors.

**Conclusions:**

Community engagement and word of mouth are vital to the successful recruitment of Latina/Latinx participants to research studies. Latinx participants are most motivated to participate by health benefits and health education, as well as altruistic aspects of research studies. Therefore, establishing mutually beneficial relationships within Latinx communities and appealing to motivations for research participation with close attention to the demographics of participants can both expand and allow for targeted recruitment efforts for this underrepresented group in research studies.

## Introduction

Latinx populations are underrepresented in research studies in the USA, despite their growing numbers [1,2]. Consequently, efforts around the recruitment of Latinx populations in research studies are especially important. Learning why Latinx populations become involved in research and how they learn about research studies can be valuable for promoting engagement in research studies.

Underrepresentation of minority populations in research studies is attributable to a variety of factors, including mistrust of medical researchers [2–8] and lack of awareness of studies or not being offered opportunities to engage in research [2,5–7]. The latter effect is particularly relevant to female participants [2]. While previous studies have investigated barriers to Latinx participation in research studies, none have identified how Latina/Latinx populations specifically learn about research studies and motivators for participating in these studies. Thus, we explored these questions in the Environment, Leiomyomas, Latinas, and Adiposity Study (ELLAS).

We hypothesized that community-based approaches (community outreach, community organization/partner) would be the most widely reported method of learning about ELLAS and that financial and health benefits would be the primary incentives for enrolling in ELLAS. We also hypothesized that the methods of learning about the study and reasons for participating would vary by sociodemographic characteristics.

## Materials and Methods

### Environment, Leiomyomas, Latinas, and Adiposity Study

ELLAS is an ongoing prospective cohort study of reproductive age Latinx women in southeast Michigan. It was approved by the University of Michigan Institutional Review Board (HUM00122341) prior to the enrollment of participants. A detailed description of the design of the study can be found in Manuel *et al*. [9]. Briefly, orientation and study visits took place at several community-based organizations and at the University of Michigan from October 2017 to March 2020. Advertisements, health care providers, and community-engaged approaches were utilized for study recruitment, which included direct mail, newsletter/email announcements, community organization listservs, and community events. Since its inception, the study has maintained a core group of staff who are bilingual and self-identify as Hispanic/Latinx. All interviews, consents, and orientations have been conducted by this group. The only-patient facing staff that have not been bilingual are the ultrasonographers, however, there has always been a bilingual staff member present during the ultrasound to facilitate communication during the ultrasound procedure.

### Study Participants

Eligible participants in ELLAS self-identified as Latina/Latinx, were between the ages of 21–50 years at the time of enrollment, could speak, read, and write in either English or Spanish, and were able to come to study visits at a community location in southeast Michigan.

### Surveys

Participants completed a demographic and health survey, including questions about education, income, health insurance, and country of birth. The four-item Short Acculturation Scale for Hispanics (SASH) [10,11] – which assessed languages read and spoken generally, at home, with friends, and language used while thinking – was used to assess acculturation. Health literacy was assessed using a single validated question asking about participants' confidence in completing medical forms [12]. Interviewers also asked research participants to identify how they heard about ELLAS and to identify their top three reasons for participating in ELLAS. The specific questions asked, and categorical response options are shown in Table 1.

**Table 1.** Survey questions about joining the Environment, Leiomyomas, Latinas, and Adiposity Study (ELLAS)

How did you hear about the study? (Select **ALL** that apply) A community organization/partner (i.e., CHASS, DHDC, La Familia) Your physician/health care provider Advertisement (flyer, announcement, newsletter) Word of mouth (friend or relative told you about it) Outreach event (Ypsilanti Heritage Festival, Latino Festival, etc.) Other, please specify: _________________________________ Don't know [*Cannot select Q1 = G with other options.*] Refuse to answer [*Cannot select Q1 = H with other options.*]
Please rank the **top 3** main reasons you joined this research study with “1” indicating the top reason, “2” indicating the second main reason, etc. _____ To receive a free health assessment _____ To learn more about women’s health _____ To contribute to scientific knowledge _____ To satisfy your curiosity about participating in a study _____ A friend/family member recommended that you join the study _____ Your physician/healthcare provider recommended that you join the study _____ To receive the financial reimbursement _____ Other, please specify: _________________________________ Don't know Refuse to answer

### Statistical Analysis

Summary statistics are presented as means and standard deviations for continuous variables and proportions for categorical variables. Since participants could select more than one option for the questions about research participation, each response was analyzed separately. Logistic regression models were used to estimate the odds ratios for sociodemographic and acculturation characteristics by participants’ selections for methods of learning about ELLAS or reasons for participation in ELLAS. All analyses were carried out using Statistical Analysis Software version 9.4 (Cary, NC).

## Results

### Sociodemographic Characteristics

633 participants were enrolled in ELLAS and 618 were eligible for analysis. Of the 633 participants enrolled, 1 was ineligible after consent due to age, 3 withdrew, 5 did not complete an orientation visit, and 6 had missing responses to reasons about research participation. Participants included and excluded from this study had comparable sociodemographic and acculturation characteristics, where available for comparison. Table 2 shows demographics and acculturation data for the 618 participants included in this analysis. Participants had a mean age of 37.5 ± 7.0 years. The majority of participants had either less than a high school degree (47.9%) or high school degree (25.6%), an annual household income of less than $30,000 (60.0%), and lacked health insurance (56.1%). In addition, most participants were born outside the USA (84.5%), with 75.7% born in Mexico. Of those born outside the USA, the mean age of immigration was 22.4 ± 7.8 years. On the Short Acculturation Scale for Hispanics, 87.6% of participants had low acculturation scores between 1 and 3 out of 5. Thirty-eight percent of participants had inadequate health literacy based on a single-validated question.

**Table 2.** Demographic and socioeconomic characteristics of participants (n = 618)

	All participants (n = 618)
Age (mean ± SD)	37.5 ± 7.02
Education	
Less than high school	296 (47.9%)
High school or GED	158 (25.6%)
Some college or associate’s degree	59 (9.5%)
Bachelor’s degree	69 (11.2%)
Master’s or doctoral degree	34 (5.5%)
Missing	2 (0.3%)
Annual household income	
$10,000 or less	74 (12.0%)
$10,001–$20,000	151 (24.4%)
$20,001–$30,000	146 (23.6%)
$30,001–$40,000	97 (15.7%)
$40,001–$60,000	53 (8.6%)
$60,001–$100,000	31 (5.0%)
More than $100,000	25 (4.0%)
Don't know	1 (0.2%)
Refuse to answer	38 (6.1%)
Missing	2 (0.3%)
Country of birth	
Caribbean Islands	4 (0.6%)
Central America	47 (7.6%)
Mexico	468 (75.7%)
South America	33 (5.3%)
United States	63 (10.2%)
Missing	3 (0.5%)
Age moved to USA^1^ (mean ± SD)	22.4 ± 7.81
Short Acculturation Scale for Hispanics (SASH)	
Lower acculturation	537 (86.9%)
Higher acculturation	77 (12.5%)
Missing	4 (0.6%)
Health insurance	
No	347 (56.1%)
Yes	265 (42.9%)
Do not know	6 (1.0%)
Health literacy	
Inadequate	234 (37.9%)
Adequate	380 (61.5%)
Do not know	1 (0.2%)
Missing	3 (0.5%)

1 Among participants born outside the USA, n = 552.

### Methods of Learning about ELLAS

“Word of mouth” through a friend or relative was the most common method of learning about ELLAS (49.0%), followed by a community “outreach event” (29.3%), “advertisement”, which includes direct mail, newsletter/email announcements, brochures, posters, and local news and radio announcements (12.8%), and “community organization/partner” (11.5%), as shown in Table 3. Learning about ELLAS through a “physician/health care provider” was the least common method (1.3%). Only 28 (4.5%) participants selected more than one option; the most common combination was “word of mouth” and “outreach event” (n = 6, 1.0%).

**Table 3.** Methods of learning about ELLAS and reasons for participating (n = 618)

	All participants (n = 618)
Method of learning about the study^1^	
Word of mouth (friend or relative)	303 (49.0%)
Outreach event	181 (29.3%)
Advertisement (flyer, announcement, newsletter)	79 (12.8%)
A community organization/partner	71 (11.5%)
Their physician/health care provider	8 (1.3%)
Other	7 (1.1%)
Missing	1 (0.2%)
All reasons for joining^1^	
Learn more about women’s health	515 (83.3%)
Receive a free health assessment	491 (79.4%)
Contribute to scientific knowledge	368 (59.5%)
A friend/family member recommended it	192 (31.1%)
Satisfy your curiosity about participating in a study	131 (21.2%)
Receive the financial reimbursement	88 (14.2%)
Their physician/healthcare provider recommended it	18 (2.9%)
Other	25 (4.0%)
Don't know	1 (0.2%)
Missing	1 (0.2%)
Primary reasons for joining	
Receive a free health assessment	313 (50.6%)
Learn more about women’s health	194 (31.4%)
Contribute to scientific knowledge	61 (9.9%)
A friend or family member recommended it	23 (3.7%)
Satisfy your curiosity about participating in a study	9 (1.5%)
Receive the financial reimbursement	6 (1.0%)
Their physician or healthcare provider recommended it	2 (0.3%)
Other	6 (1.0%)
Don't know	1 (0.2%)
Missing	3 (0.5%)

1 Participants could choose more than one response.

**Fig. 1.**
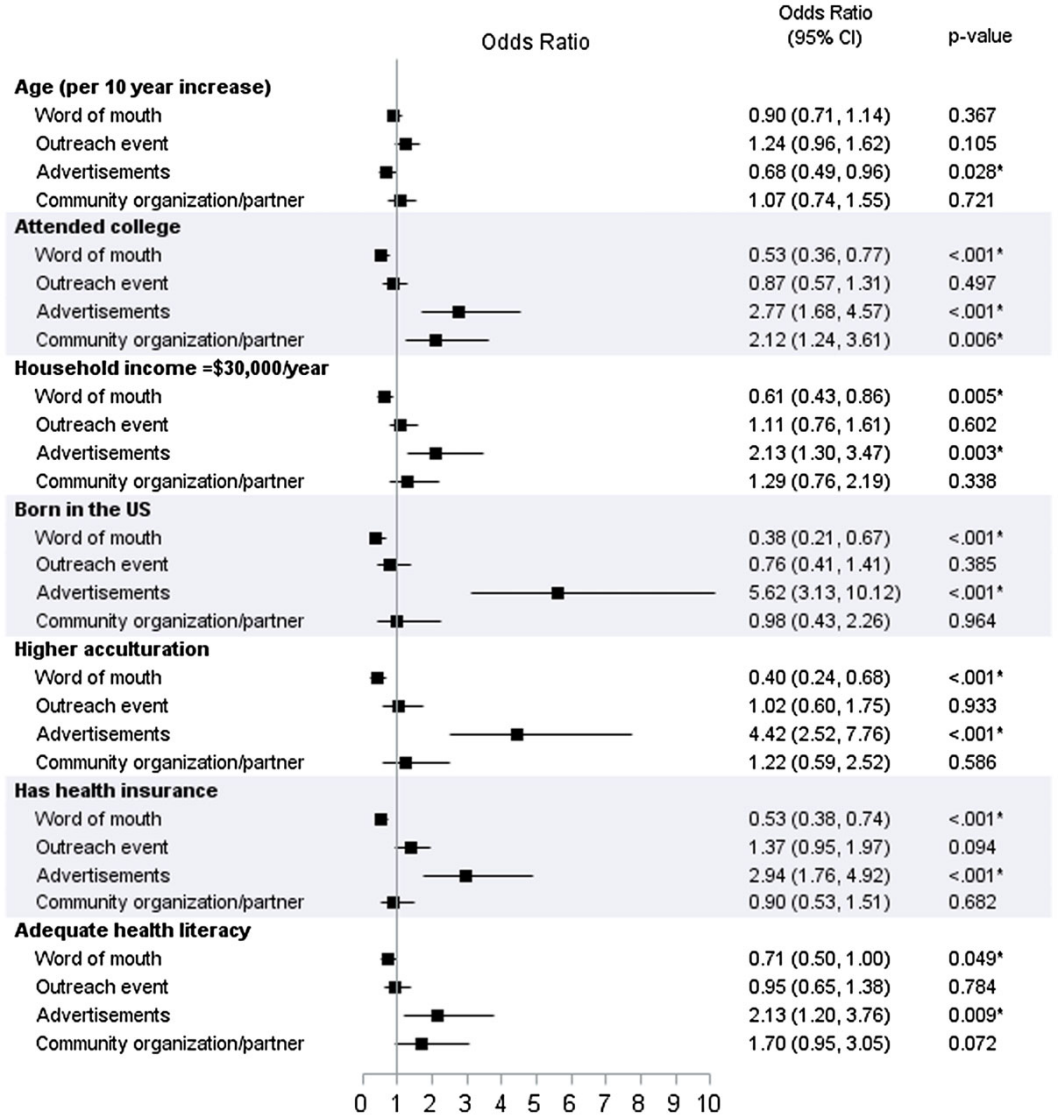
Forest plot illustrating the results from logistic regression models for methods of learning about the Environment, Leiomyomas, Latinas, and Adiposity Study (ELLAS).

Demographic and socioeconomic characteristics by the top four methods of learning about the study are shown in Table 4 and odds ratios from logistic regression models for each method are shown in Fig. 1. “Word of mouth” was associated with not attending college and household income <$30,000/year, being born outside the USA, lower acculturation, not having health insurance, and inadequate health literacy. In contrast, participants who listed “advertisement” as their method of learning about the study were younger, were more likely to have attended college, and have a household income ≥$30,000/year, were more likely to be born in the USA and be more acculturated, had health insurance, and had adequate health literacy. None of the sociodemographic variables were associated with “outreach event” as participants’ method of learning, and only attending college was associated with “community organization/partner”.

**Table 4.** Demographic and socioeconomic characteristics by method of learning about the Environment, Leiomyomas, Latinas, and Adiposity Study (ELLAS) (n = 618)

	N	Word of mouth (n = 301)	Outreach event (n = 180)	Advertisements (n = 77)	Community organization/ partner (n = 70)
Age (mean ± SD)		37.2 ± 6.96	38.3 ± 6.55	35.9 ± 8.09	37.9 ± 7.37
Education					
High school/GED or less	454	240 (52.9%)	138 (30.4%)	42 (9.3%)	42 (9.3%)
At least some college	162	62 (38.3%)	43 (26.5%)	36 (22.2%)	29 (17.9%)
Annual household income					
<$30,000	371	199 (53.6%)	106 (28.6%)	37 (10.0%)	38 (10.2%)
≥$30,000	206	85 (41.3%)	62 (30.1%)	39 (18.9%)	27 (13.1%)
Born in the USA					
No	552	284 (51.4%)	165 (29.9%)	53 (9.6%)	64 (11.6%)
Yes	63	18 (28.6%)	16 (25.4%)	24 (38.1%)	7 (11.1%)
Acculturation					
Lower acculturation	537	278 (51.8%)	157 (29.2%)	52 (9.7%)	61 (11.4%)
Higher acculturation	77	24 (31.2%)	23 (29.9%)	25 (32.5%)	10 (13.0%)
Health insurance					
No	347	193 (55.6%)	92 (26.5%)	28 (8.1%)	40 (11.5%)
Yes	265	107 (40.4%)	86 (32.5%)	51 (19.2%)	31 (11.7%)
Health literacy					
Inadequate	234	125 (53.4%)	71 (30.3%)	18 (7.7%)	21 (9.0%)
Adequate	380	176 (46.3%)	109 (28.7%)	60 (15.8%)	50 (13.2%)

### Reasons for Participating in ELLAS

The three overall most common reasons for participating in ELLAS were “to learn more about women’s health” (83.3%), “to receive a free health assessment” (79.4%), and “to contribute to scientific knowledge” (59.5%), as shown in Table 2. When participants were asked to rank their top three reasons, “to receive a free health assessment” (50.6%) and “to learn more about women’s health” (31.4%) were the most common primary reasons for participating. Only 14.2% cited “to receive financial reimbursement” as one of their top three reasons, with a mere 1.0% listing it as their primary reason. The least common reason chosen as any of the top three reasons was their “physician or healthcare provider recommended [joining]” (2.9%). The most common combination of responses was “to receive a free health assessment,” “to learn more about women’s health,” and “to contribute to scientific knowledge” (n = 200, 32.4%).

**Fig. 2.**
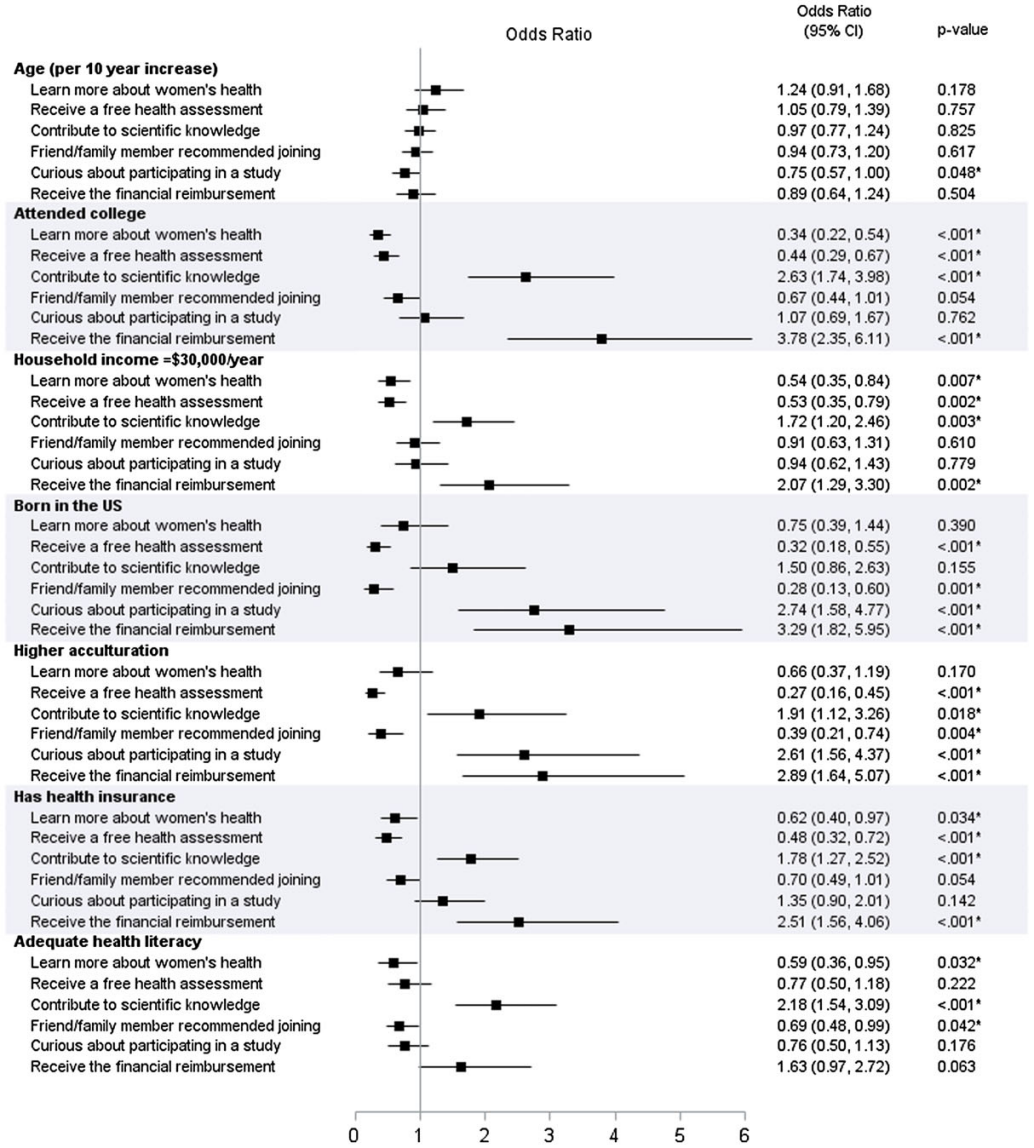
Forest plot illustrating the results from logistic regression models for reasons for joining the Environment, Leiomyomas, Latinas, and Adiposity Study (ELLAS).

Demographic and socioeconomic characteristics for the top six reasons participants joined ELLAS are shown in Table 5 and odds ratios from logistic regression models for each reason for participating are in Fig. 2. Not attending college and household income <$30,000/year, lack of health insurance, and inadequate health literacy were associated with participants joining “to learn more about women’s health.” Participants joining “to receive a free health assessment” also were more likely to not have attended college and have household income <$30,000/year, be born outside the USA and have lower acculturation, and not have health insurance. Participants who listed “to contribute to scientific knowledge” as one of their reasons were more likely to have attended college and have household income ≥$30,000/year, higher acculturation, health insurance, and adequate health literacy. Joining ELLAS because “a friend/family member recommended [it]” was associated with being born outside the USA, lower acculturation, and inadequate health literacy. Participants who wanted “to satisfy curiosity about participating in a study” were more likely to be younger, born in the USA, and have higher acculturation. Finally, participants who cited “to receive financial reimbursement” as one of their reasons were more likely to have attended college, have household income ≥$30,000/year, be born in the USA, have higher acculturation, and have health insurance.

**Table 5.** Demographic and socioeconomic characteristics by all reasons for joining the Environment, Leiomyomas, Latinas, and Adiposity Study (ELLAS) (n = 618)

	N	Learn more about women’s health	Receive a free health assessment	Contribute to scientific knowledge	Friend/family member recommended joining	Curious about participating in a study	Receive the financial reimbursement
Age (mean ± SD)		37.7 ± 6.99	37.5 ± 6.95	37.5 ± 6.89	37.3 ± 7.04	36.3 ± 7.56	37.3 ± 7.17
Education							
High school/GED or less	454	398 (87.7%)	380 (83.7%)	244 (53.7%)	152 (33.5%)	93 (20.5%)	42 (9.3%)
At least some college	162	115 (71.0%)	110 (67.9%)	122 (75.3%)	40 (24.7%)	37 (22.8%)	46 (28.4%)
Annual household income							
<$30,000	371	319 (86.0%)	308 (83.0%)	202 (54.4%)	121 (32.6%)	82 (22.1%)	43 (11.6%)
≥$30,000	206	159 (77.2%)	148 (71.8%)	139 (67.5%)	64 (31.1%)	44 (21.4%)	43 (20.9%)
Born in the US							
No	552	464 (84.1%)	453 (82.1%)	323 (58.5%)	184 (33.3%)	105 (19.0%)	67 (12.1%)
Yes	63	49 (77.8%)	37 (58.7%)	43 (68.3%)	8 (12.7%)	25 (39.7%)	21 (33.3%)
Acculturation							
Lower acculturation	537	454 (84.5%)	445 (82.9%)	310 (57.7%)	179 (33.3%)	100 (18.6%)	64 (11.9%)
Higher acculturation	77	58 (75.3%)	44 (57.1%)	56 (72.7%)	13 (16.9%)	30 (39.0%)	23 (29.9%)
Health insurance							
No	347	298 (85.9%)	293 (84.4%)	187 (53.9%)	118 (34.0%)	64 (18.4%)	33 (9.5%)
Yes	265	212 (80.0%)	192 (72.5%)	178 (67.2%)	73 (27.5%)	66 (24.9%)	54 (20.4%)
Health literacy							
Inadequate	234	205 (87.6%)	194 (82.9%)	113 (48.3%)	83 (35.5%)	55 (23.5%)	25 (10.7%)
Adequate	380	307 (80.8%)	294 (77.4%)	253 (66.6%)	109 (28.7%)	76 (20.0%)	62 (16.3%)

## Discussion

This study was the first to investigate motivations for participating in and methods of learning about medical research studies in a reproductive-aged female Latinx population. Approximately half the participants in ELLAS learned about the study through “word of mouth.” The most common primary reason for participating in ELLAS was “to receive a free health assessment,” followed by “to learn about women’s health.” Of note, we found that certain socioeconomic characteristics and social determinants of health, including educational attainment, household income, having health insurance, acculturation, and health literacy, were associated with different methods for how reproductive age Latinx participants learned about studies and different motivations for participating in studies.

Many of the participants in ELLAS learned about the study through “word of mouth” from a family member or friend, illustrating the importance of informal community and individual networks for recruitment of Latinx populations in research studies. We specifically found that having a lower education and lower income, lack of health insurance, lower acculturation, being born outside the USA, and inadequate health literacy were more strongly associated with learning about ELLAS through “word of mouth.” In a population where the majority of people are of lower socioeconomic status, advertisements may play less of a role in visibility of research studies.

Our findings are similar to other studies investigating recruitment efforts to increase enrollment of underrepresented groups to research studies. Horowitz et al found that community-partnered approaches such as recruiting at public events and enlisting participants to recruit people in their community were more successful than clinician referrals or advertisements [13]. Other studies have confirmed that engagement of community groups and community-focused recruiting practices are the most effective [13–21]. Community-partnered approaches appear to be more effective in successful recruitment of traditionally underrepresented groups into research.

Prior studies demonstrate that study participants in general elect to partake in research for a variety of reasons, including as a way of receiving care [22–25], as well as a willingness to contribute to the understanding of diseases to which they have personal connections [26], or as a way of helping others [23–25,27–32]. A prior study that specifically included minority cardiovascular research participants by Gill et al found that the primary motivator for research participation was for a health and heart checkup [33]. In women with Systemic Lupus Erythematosus engaging in research studies, learning more about their condition was a reason for participating in the studies [30]. This is similar to the desire of ELLAS participants to learn more about women’s health, indicating that future study recruitment can benefit from emphasizing health education.

We found demographic and socioeconomic associations with specific reasons for participating in research. Participants who were interested in learning about women’s health were more likely to have a lower education and income, have no health insurance, and have inadequate health literacy. This may be because participants with higher education had more exposure to women’s health subjects through education and/or exposure to the healthcare system. Additionally, those who were motivated by receiving a free health assessment were more likely to have lower income and no health insurance, indicating that health benefits play an important role in research participation for people who have less access to resources. Conversely, those who picked “financial reimbursement” as a motivator for participating in ELLAS were more likely to have a higher education, income above $30,000, have health insurance, and have higher acculturation. This relationship seems paradoxical, as participants with lower income were less interested in financial reimbursement than those with a higher income. Prior research has not fully explored the impact of various demographic and socioeconomic factors on people’s decision to participate in research in different populations. However, there are several studies that have shown that age, health literacy, sex, and education levels are broadly associated with study participation [34–36].

Overall, only 14.2% of participants in ELLAS listed “financial reimbursement” as one of their top three reasons for participating in the study. A study investigating motivations for participating in a school-based parenting program in Latinx and low-income communities found that intrinsic motivation and benefits from the program outweighed the incentive of receiving extra money to attend these sessions [37]. One study that investigated motivations of German women for participation in a clinical trial for menstrual pain found financial incentives less important than an interest in finding alternative solutions to dealing with pain and furthering research in a field that affects participants [38]. These studies indicate that altruistic and health benefits outweigh financial incentives for research participation for female participants, although none have compared these specific motivations between participants of different incomes and demographics. Our findings demonstrate that financial incentives may not play an important role in lower socioeconomic Latinx populations in the way that educational, altruistic, and health benefit motivations do.

ELLAS focuses on a targeted group of individuals – Latina/Latinx communities in Southeast Michigan. Although the demographics in our study population closely match that of the Latinx/Hispanic population in the USA [39], it is possible that our findings may not be generalizable to other Latinx communities. Another potential weakness of this study is that participants were given options to choose from rather than an open-ended questionnaire, which could have led to answers that were not listed in the answer options we did not anticipate. Future studies should investigate the association between different demographics and motivations/methods of learning about research more broadly, to aid in recruitment of different groups in research studies.

Strengths of this study include utilizing a community advisory board (CAB), a community-based participatory approach to recruitment design, as well as bilingual staff, which allowed ELLAS to be more accessible. The CAB was composed of community members who provided insights during the development of ELLAS protocols from inception, thus ensuring the community’s interests were represented and that the study was administered in a culturally appropriate and equitable fashion. Community partners have the trust of the community through pre-existing longstanding relationships, and participants likely feel more comfortable engaging in research as a result of the involvement of these partners. The Principal Investigator (EEM) and study team spent more than 18 months working with and learning from CAB and other community members to ensure that the foundation of mutual trust and respect was present before initiating recruitment. Additionally, cultural background was considered throughout the study and is reflected in the events that were provided, questions used in the surveys, incentives given, and the special attention that was given to help participants feel at ease. The ELLAS study site was based in the community in which participants lived, making ELLAS more visible and convenient to participants than if it were located at an academic center. Another strength of our study was that we investigated demographics that are associated with different methods of learning about and reasons for participating in research studies, which can help future studies focus on recruitment efforts targeting the needs of Latina/Latinx populations.

In summary, this study of more than 600 reproductive age Latinx participants found that “word of mouth” and “outreach events” are the most successful approaches to reach Latinx communities for study recruitment, and that healthcare benefits and the altruistic and educational nature of this study appealed the most to Latinx study participants overall. Furthermore, we found that socioeconomic status is correlated with different methods of learning about and motivations for participating in research studies, which can aid in targeted recruitment for specific segments of the Latinx community for future research studies. Our findings can help researchers select the most targeted and high yield approaches for recruitment and engagement, given their ideal recruitment population.
